# Age, sex and seasonal differences in COVID-19 viral load: a Lebanese cross-sectional study

**DOI:** 10.1186/s12879-025-11955-7

**Published:** 2025-11-03

**Authors:** Elie Charro, Karolina Jaalouk, Fatima Kourani, Youmna Mrad, Fadi Fakih, Chadi Fakih

**Affiliations:** 1 Faculty of Medical Sciences, Lebanese University, Hadath, Lebanon; 2 Al Hadi Medical Center Director, Beirut, Lebanon

**Keywords:** COVID-19, CT-values, Sex, Age, Seasonal variation

## Abstract

**Background:**

Coronavirus disease 2019 (COVID-19) is a respiratory infection caused by a virus known as severe acute respiratory syndrome coronavirus 2. It has resulted in more than 6 million deaths worldwide and continues to impact our daily lives. Diagnosis of the disease relies on determining the CT (Cycle Threshold) value through reverse transcription polymerase chain reaction with a higher CT-Value indicating a lower viral load. While COVID-19 has been studied in terms of pathophysiology, symptoms, and treatment, fewer large-scale studies have addressed how viral load varies by age, sex, and season. This study aims to illustrate if sex, age, and seasonal changes have an impact on the viral load of COVID-19 within an initial Lebanese cross-sectional study.

**Methods:**

Data for our study was gathered from the Al-Hadi Medical Center since the onset of the pandemic. A total of 686,739 participants were included in our analysis. Age, sex, and seasonal variation were extracted from the available data. Individuals under the age of 1 were excluded from the study.

**Results:**

The predictors for a positive PCR were being a female, being a child or a senior, and testing during a cold and humid season. Children had a higher CT value compared to seniors. Linear regression showed that an increase of 1 year in age is mirrored by a decrease of 0.013 in CT value. Concerning sex, females have an average of 0.149 lower CT value than males. Finally, the CT value is higher by an average of 1.392 in cold and rainy seasons compared to hot and dry ones.

**Conclusion:**

Sex, age, and seasonal variations are modest predictors of the viral load of COVID-19. All these demographic and seasonal factors may affect the viral load and should be taken into consideration when implementing infection control strategies and clinical management in SARS-CoV-2-positive patients.

## Introduction

Coronavirus disease 2019 (COVID-19), first identified in Wuhan, China, in December 2019, quickly spread worldwide. Lebanon reported its first confirmed case on February 21, 2020, and has since experienced multiple waves of infection amidst profound social, economic, and healthcare challenges [1–3]. By March 2023, over 1.2 million confirmed cases and more than 10,000 deaths had been reported in the country [4]. COVID-19 severity and clinical outcomes vary considerably between individuals. Several factors, including age, sex, and comorbidities, have been proposed to explain this heterogeneity [5]. Reverse transcription polymerase chain reaction (RT-PCR) remains the gold standard for diagnosis, with the cycle threshold (CT) value serving as an indirect indicator of viral load [6–8]. Higher viral loads, reflected by lower CT values, have been associated with greater disease severity and mortality [6, 7]. Despite extensive research, the influence of demographic and temporal factors on viral load remains insufficiently explored, particularly in Middle Eastern populations.

Concerning the pathophysiology of COVID-19, viral entry is firstly mediated by binding of the virus to ACE-2 receptors [9], which highlights that higher ACE-2 receptors increases lung vulnerability by increasing pulmonary vessel permeability and inflammation [10]. This fusion is facilitated by androgen-mediated host proteases, such as TMPRSS2 (transmembrane protease serine 2, a serine 2 encoding gene) [11, 13].

Evidence suggests that both age and sex influence immune responses to SARS-CoV-2. Older individuals demonstrate diminished antiviral immunity and decrease in the efficiency of particle clearance, hence they are more likely to test positive for COVID-19with RT-PCR [14, 16]. While sex differences in immune regulation (such as X-chromosome inactivation (XCI)) and hormone-mediated mechanisms (such as elevated androgen levels in males) have been linked to variations in disease outcomes showing that women suffered less from mortality rates compared to men [9–18]. Seasonal fluctuations may further shape viral transmission and host responses, with temperature and humidity shown to affect respiratory virus dynamics, as droplet transmission is relatively increased by humidity and other factors like social distancing measures and age are thought to still have the more important effect on the transmission rate [19–22]. However, data on the interplay of age, sex, and seasonal variation with COVID-19 viral load remain limited.

The primary objective of this study was to assess the effect of sex, age, and seasonal differences on SARS-CoV-2 viral load in a first emerging Lebanese cohort. The secondary objectives were to evaluate seasonal variations in viral load across age and sex groups, and assess age-related differences in viral load within each sex group.

## Materials and methods

### Study design

In this retrospective study, electronic medical records of COVID-19 patients were collected through the utilization of the LIS Software from a private laboratory center called ALHadi Medical Center, commencing from March 2020 till May 2023. Laboratory Information System (LIS) is specialized healthcare software designed to manage and organize patient data pertaining to laboratory testing and related processes.

### Participants

Using Cochran’s Equation, with a precision level of 5%, a confidence level of 95%, and an estimated proportion of 50%, considering an unlimited sample size, the study needed to be performed on a minimum of 385 participants. However, after collecting data from the medical center, we had a total of 686,739 participants. Data for participants less than 1 year were excluded from the study.

### Data collection and measurements

Healthcare workers at Al Hadi Center collected specimens from suspected COVID-19 patients, despite being symptomatic or not, while adhering to safety protocols issued by international protocols. Nasopharyngeal swabs were collected and placed in 2–3 mL sterile normal saline. Viral RNA was extracted using an automated microbead-based system (input, 400 µL; elution, 50 µL) following the addition of an exogenous internal control to monitor extraction efficiency. Reverse transcription quantitative PCR (RT-qPCR) was performed targeting the SARS-CoV-2 nucleocapsid (N), envelope (E), and RNA-dependent RNA polymerase (RdRP) genes, alongside amplification of an endogenous control and the internal control to assess sample quality. Samples were classified as positive if all three viral targets were detected.Following the Lebanese Ministry of Public Health guidelines and the internal diagnostic policy of Al Hadi Medical Center, a CT value < 35 was considered positive throughout the study period. This threshold was chosen to maximize sensitivity in clinical practice and was consistently applied across all samples. Participants were categorized as positive or negative cases and further analyzed quantitatively based on CT values.

### Variables

Age was calculated from the difference between date of birth and sample submission date. Sex comparison was made between males and females. For seasonal variation, Lebanon’s Mediterranean climate was characterized by hot, dry summers (April-September) and cold, rainy winters (October-March). Data were grouped into hot/dry and cool/rainy seasons accordingly. Because day-level meteorological data (temperature, relative humidity) were not available in the laboratory information system, we operationalized season as a binary proxy (cold/rainy vs. hot/dry) defined a priori based on the local climate. The study period (March 2020–May 2023) encompassed multiple phases of the pandemic, including the initial wave and subsequent variant-associated surges (Alpha, Delta, Omicron). While the calendar year of testing was recorded and is presented descriptively, variant-specific or vaccination-related data were not available in the LIS and therefore could not be incorporated into the analysis.

### Data analysis

The univariate, bivariate (chi-square, ANOVA, Turkey post-hoc) and multivariate analysis was conducted using IBM Statistical Package for the Social Sciences (SPSS) version 27.0. The first part of the analysis, we studied all the data acquired no matter if the PCR test was positive and negative.

A descriptive analysis was started and the variables were presented depending on their type. The continuous variables were shown as the mean, median, frequency and standard deviation, as an example (Age, CT value). On the other hand, categorical variables were presented as proportions and frequency, like sex and test result. Bivariate analysis was used to assess the correlation between the result of the PCR test if it is positive or negative with the three variables: age, sex and seasonal variation. The analysis was done using t-test and chi-square test. Multivariate analysis was done using logistic analysis to assess the predictors of a positive PCR test. Sex, age and seasonal variations were considered as predictors. Missing results were excluded, so the number of cases included in the multivariate analysis is *n* = 655,035 (95.4% of the total).

For the second part, all negative results were excluded and all the multivariate and bivariate analyses were done on PCR positive individuals. In this section, raking was done to give male and females equal statistical weight. Bivariate analysis was done to assess the correlation between CT value and each of the following variables: age, sex and season. In order to study more the effect of predictors on CT value, multivariate linear regression was done while changing the constant variable. This includes: CT value, age, sex and seasonal variation. The tests used were: chi-square, t-test, linear regression, and Non parametric Kruskal-Wallis test.

Significance was set at *p* < 0.05.

## Results

In this study, the total number of participants was 686,739 of whom 52.7% were males and 47.3% were females. Most of the participants presented during the cold and humid seasons (59.1%) and the majority (83.4%) of total participants tested negative. The year 2021 was found the have the highest number of tests done (49.9%). Mean CT-value was 24.38 ± 4.77 and mean age was 27.24 ± 17.12, ranging from 1 to 110 years, with adults comprising 78.6% and seniors 7.2% of the sample. For continuous variables (age and CT value), skewness and kurtosis values ranged between − 1 and + 1, indicating normality. Table 1 presents the descriptive analysis.

**Table 1:** Descriptive analysis of participants who presented for the test

Variable		*N* (%)
Sex	Female Male	324 659 (47.3%) 362 080 (52.7%)
Season	Hot & Dry (Months 4–9) Cold & Humid (10 − 3)	280 861 (40.9%) 405 878 (59.1%)
Age Group	Children (< 18 years) Adults (18–64 years) Seniors (> 64 years) Missing	65 956 (9.6%) 539 512 (78.6%) 49 567 (7.2%) 31 704 (4.6%)
Year	2020 2021 2022 2023	138 108 (20.1%) 342 496 (49.9%) 197 682 (28.8%) 8 453 (1.2%)
PCR Result	Negative (CT ≥ 35) Positive (CT < 35)	574 657 (83.4%) 112 082 (16.6%)

### Bivariate analysis for PCR results

Table 2 shows that women are more likely to have a positive PCR result than men. Children (adjusted residual 30.4) and Seniors (adjusted residual 10) also had higher odds of a positive PCR result compared to adults (adjusted residual − 30.9). Positive PCR results occur more often in cold, humid seasons than in hot, dry seasons.

**Table 2:** Association between the result of PCR test with sex, age groups and season

Variables		Positive	Negative	Pearson Chi-Square
Sex	Female Male	58 189 (17.9%) 58 893 (14.9%)	266 470 (82.1%) 308 187 (85.1%)	1157.42***
Age	Children (< 18 years) Adults (18–64 years) Seniors (> 64 years)	13 677 (20.7%) 85 846 (15.9%) 9 005 (18.2%)	52 279 (79.3%) 453,666 (84.1%) 40,562 (81.8%)	1088.90***
Season	Hot & Dry (Months 4–9) Cold & Humid (10 − 3)	27 896 (9.9%) 84 186 (20.7%)	252 965 (90.1%) 321,692 (79.3%)	14201.56***

*** *p* < 0.001

### Multivariate analysis for PCR results - logistic analysis

PCR results were taken as a dependent variable. Logistic analysis was done (Table 3) to assess the predictors of positive PCR test. The concluded predictors are being female (1.25x), being a child (1.36x), being a senior (1.14x), and testing during the cold and humid seasons (2.35x).

**Table 3:** PTG multivariate analysis – logistic analysis for PCR results

	Beta (Exp Beta)	Standard Error	*p*-value
Age: Children Adults Senior	0.306 (1.358) Ref 0.132 (1.141)	0.010 0.012	< 0.001 < 0.001
Sex Female Male	0.222 (1.249) Ref	0.007	< 0.001
Season Cold & Rainy Hot & Dry	0.853 (2.347) Ref	0.008	< 0.001
Constant	-664.959 (0)	9.635	< 0.001

All subsequent analyses were conducted exclusively on positive cases (*n* = 112,651). Prior to analysis, raking was applied to balance the sex distribution using weights of 1.04 for males and 0.96 for females, resulting in an equal-weighted frequency of 56,041 (50%) for both sexes (original distribution: 45.4% males, 54.6% females).

### CT value in positive cases: bivariate analysis

As per Table 4, females who tested positive had a lower mean CT value (24.33 ± 4.79) compared to males (24.44 ± 4.75). Concerning seasonal variation, the mean CT value was higher in cold and rainy season (24.73 ± 4.67) compared to hot and dry ( 23.35 ± 4.93).

**Table 4:** Correlation between CT value and the two variables: sex and season

	Mean CT ± SD	T-test	Mean Diff	95% CI UL	95% CI LL
Sex Female Male	24.33 ± 4.79 24.44 ± 4.75	4.052***	0.11 ± 0.02	0.17	0.05
Season Hot & Dry Cold & Rainy	23.35 ± 4.93 24.73 ± 4.67	-41.246***	-1.38 ± 0.03	-1.32	-1.45

*** *p* < 0.001

Pearson correlation between CT Value and age was negative. Pearson coefficient of -0.052 (*p* < 0.001). Since variances were not homogeneous (Levene’s test *p* < 0.001), a non-parametric Kruskal-Wallis test was used and showed a significant difference in CT values across age groups (*p* < 0.001). Bonferroni post-hoc analysis confirmed that all pairwise comparisons were significant (*p* < 0.001). Children had the highest CT value (24.75 ± 4.67) while seniors had the lowest (23.88 ± 4.91). This reflects that seniors have a higher viral load.

### CT value in positive cases: multivariate linear regression

To further evaluate the predictors of CT value, a multivariate analysis was done with CT value as the dependent variable (Table 5). Results showed a negative association with age, with a decrease of 0.013 units with a 1-year increase in age (*p* < 0.001). Females had a lower CT value by 0.149 compared to males (*p* < 0.001). In the cold and rainy season, the CT value was significantly higher compared to the hot and dry season (*p* < 0.001). The overall model was statistically significant; however, the explanatory power was limited, with an R² of 0.019, indicating that age, sex, and season together accounted for only a small proportion of the variance in CT values.

**Table 5:** Multivariate linear regression between CT value as a constant with age, sex and season

	UnStd. Beta	Std. Error	Std. Beta	t	*p*-value
Constant	23.888	0.044		543.624	< 0.001
Age	-0.013	0.001	-0.047	-15.729	< 0.001
Sex Female Male	-0.149 Ref	0.029	-0.016	-5.191	< 0.001
Season Cold & Rainy Hot & Dry	1.392 Ref	0.033	-0.126	41.696	< 0.001

R-square 0.019

### CT value in positive cases-multivariate linear regression: sex as a selection variable

To further examine the effect of age and seasonal variation on CT value across sexes, a multivariate linear regression was performed separately for males and females (Table 6). Age had a slightly stronger negative association with CT value in females (β = − 0.014) compared to males (β = − 0.011). A similar pattern was observed for seasonal variation, with a slightly higher increase in CT value during the cold and rainy season in males compared to females.

### CT value in positive cases-multivariate linear regression: season as a selection variable

The same regression model was applied, this time stratified by season (Table 6). In the hot and dry season, the difference in CT values between sexes was not statistically significant. Additionally, the effect of age on CT value was reduced compared to the cold and rainy season, with the beta coefficient decreasing from − 0.014 in the cold and rainy season to − 0.008 in the hot and dry season.

### CT value in positive cases-multivariate linear regression: age as a selection variable

To assess the effect of sex and season on COVID-19 viral load across different age groups, multivariate linear regression was performed stratified by age (Table 6). Among children, sex differences in CT values were not statistically significant (*p* = 0.103). In contrast, sex had a significant effect among adults and seniors (*p* < 0.001), with a stronger effect observed in seniors (β = − 0.287). Seasonal differences in CT values were significant across all age groups, with the greatest effect in children (β = 1.515), followed by adults (β = 1.405), and the smallest effect observed in seniors (β = 1.165).

**Table 6:** Multivariate linear regression taking each variable as the selection variable

Stratification	Subgroups	Predictor	UnStd. Beta	Std. Error	Std. Beta	*p*-value
By Sex	Male	Constant	23.796	0.058		< 0.001
		Age	-0.011	0.001	-0.041	< 0.001
		Cold & Rainy Hot & Dry	1.432 Ref	0.047	0.131	< 0.001
	Female	Constant	23.832	0.060		< 0.001
		Age	-0.014	0.001	-0.054	< 0.001
		Cold & Rainy Hot & Dry	1.352 Ref	0.048	0.120	< 0.001
By Seasonal Variation	Cold & Rainy	Constant	25.344	0.040		< 0.001
		Age	-0.014	0.001	-0.054	< 0.001
		Female Male	-0.166 Ref	0.033	-0.018	< 0.001
	Hot & Dry	Constant	23.686	0.076		< 0.001
		Age	-0.008	0.002	-0.029	< 0.001
		Female Male	-0.99 Ref	0.061	-0.010	0.103
By Age Groups	Children	Constant	23.485	0.101		< 0.001
		Female Male	0.045 Ref	0.079	0.005	0.569
		Cold & Rainy Hot & Dry	1.515 Ref	0.104	0.123	< 0.001
	Adults	Constant	23.437	0.036		< 0.001
		Female Male	-0.166 Ref	0.032	-0.017	< 0.001
		Cold & Rainy Hot & Dry	1.405 Ref	0.037	0.128	< 0.001
	Seniors	Constant	23.192	0.113		< 0.001
		Female Male	-0.287 Ref	0.103	-0.029	0.005
		Cold & Rainy Hot & Dry	1.162 Ref	0.116	0.105	< 0.001

## Discussion

### Age and CT value

In our study, we found that children (< 18 years old) and seniors (>64 years old) had higher odds of a positive PCR result compared to adults (18–64 years old). Seniors were also found to have the lowest CT value (23.88 ± 4.91), followed by adults (24.40 ± 4.76) and then children (24.75 ± 4.67), reflecting an increase in viral load with an increase in age. Comparably, other studies have reported conflicting findings regarding the relationship between age and viral load. For example, To et al. observed higher viral loads in younger children using oropharyngeal saliva samples, while others reported a trend toward higher viral loads in older patients. However, these studies used different specimen types and were based on small samples, limiting direct comparison to our nasopharyngeal-swab-based analysis [6, 23]. This shows that the correlation between age and viral load is extremely unclear, especially in the setting of multiple studies that haven’t found any correlation at all [14, 24].

When it comes to the correlation of age with disease severity, it is well established that age is a risk factor for the development of severe COVID-19 infection. One study found that older individuals, above 60 years of age, were at higher risk of developing critical illnesses [25], while another found that patients above the age of 70 were more likely to be admitted to the ICU [15]. An important aspect of these findings was the overall effect of comorbidities such as hypertension, or COPD; however, the increase in age itself showed to have a far more important effect on disease severity than the comorbidities [17]. And while our study lacked data relating to a patient’s comorbidities, these results appear to be coherent with our findings of increased viral load with an increase in age.

Immunosenescence, or age-related changes in the immune system, was proposed as one of the reasons why viral loads are higher in older patients [26], such as the acquisition of senescence-associated secretory phenotype (SASP) in cells, allowing them to secrete higher levels of proinflammatory cytokines [27], as well as an increase in the expression of viral entry receptor genes and TMPRSS2 [28]. Other changes that were mentioned in the literature include decreased expression of ACE-2 in the lungs [29]. On one hand, ACE-2 by itself has been proven to prevent severe lung damage, and converts angiotensin II into angiotensin 1–7, which promotes the expression of anti-inflammatory cytokines and inhibits the secretion of proinflammatory ones [28].

A higher viral load can also be attributed to a reduction in the number of ciliated cells lining the airways [16], and the increase in cellular aging and deterioration [17], which decreases the lung maturity. Also, age-related decline in respiratory tract alveolar macrophages (AMs) is accompanied by functional deficits such as phagocytosis and scavenging impairment [30].

### Sex and CT value

A study done in Saudi Arabia [24] shows higher viral loads in females, while other studies demonstrated similar viral loads between both sexes. Compared to our study, 17.9% of females tested positive for COVID, compared with 14.9% of men. And when only positive PCR results were considered, men were found to have higher CT values. In our dataset, females demonstrated slightly lower CT values than males, which indicates a marginally higher viral load. This was statistically robust in our large sample but unlikely to be clinically directive at the individual level. This appears to contrast with the broader literature showing that men experience higher rates of severe disease and mortality. These observations suggest that viral load alone is not sufficient to explain sex differences in COVID-19 outcomes. Some biological and immunological mechanisms — such as androgen-driven TMPRSS2 expression, endothelial injury, and differential immune responses — likely account for the worse outcomes in males despite comparable or lower viral loads. Other reasons might be related to the differences in testing behavior/access (e.g., earlier testing after exposure, mandated screening in specific groups), and unmeasured timing relative to symptom onset could explain the observed CT distributions.

Concerning the severity of infection, men have a higher risk of developing severe disease and mortality than women. While our study only assessed viral load, not clinical outcomes, these broader epidemiologic findings provide important context. These findings were attributed to an androgen-dependent increase in TMPRSS2 expression [31] and, in turn, an increase in ACE-2 expression. Both of which facilitate viral entry into the cell and increase endothelial damage and inflammation by increasing endothelial permeability and promoting leukocyte adhesion and, hence, the development of thrombosis and pulmonary edema, causing ARDS [32]. The stronger immune system in females can be attributed to the escape of X-chromosome inactivation of the ACE-2 gene [33], which ensures greater adaptability and plasticity [34] and makes SARS-COV-2 more tolerable [10]. Overall, our findings suggest that while sex-based differences in CT values exist, they do not explain the differences in disease severity described in the literature. This emphasizes the need for further research that integrates both host and viral factors.

### Seasonal variation and CT value

Our results showed that CT value was higher in cold and rainy season compared to hot and dry, indicating a lower viral load in cold/rainy season. Since COVID-19 is a droplet transmitted virus, where both humidity and temperature affect the viral stability within the droplet. Increased relative humidity leads to lower rates of transmission [35] as it inhibits droplet evaporation and makes it settle more quickly [36]. Moreover, increased temperature also leads to lower rates of transmission. The combined effect of temperature and humidity accounts for the seasonal variations observed.

Several biological mechanisms have been proposed in the literature to explain seasonality in respiratory infections, including impaired mucociliary clearance with cold air, modulation of interferon-stimulated genes (ISGs) by humidity, and altered host susceptibility due to environmental factors [36, 37]. However, these remain hypothetical and speculative and were not directly evaluated in our study.

On the other hand, the relationship between seasonal variations and CT value were, to the best of our knowledge, lacking, and our study aimed to find if such a relationship exists. Our study showed that positive PCR results occur more often in cold, humid seasons rather than in hot, dry seasons, with lower mean CT values during dry and hot seasons and higher means in cold and humid seasons. These results could be an indicator of higher viral load in dry and hot seasons. However, further research is needed to clarify this relationship.

### Interactions between age, sex, seasonal variations and CT value

Our analysis revealed that in males, age demonstrated a significantly stronger association with CT values, and that CT value differences between males and females were more pronounced in adults and seniors than in children. The effect of age on CT value is double in cold and rainy seasons compared to hot and dry seasons. The opposite was noted when we observed seasonal differences comparing age groups. While children and adults showed a noticeable increase in CT values during the cold and rainy seasons, this seasonal change was less evident in the elderly population.

The results pertaining to males having stronger age-CT value association are suggestive of the combined effect of both an increase in age and being male on CT value, both of which have been proposed in the literature as potentially more efficient viral entry sites due to higher TMPRSS2 expression [16, 31]. As for the sex differences being more pronounced in adults and the elderly, this can be due to the presence of risk factors, including but not limited to a reduction in ciliary lining, shrinkage of alveolar surface area, a decrease in lung elasticity, dilation of airway spaces, and a decline in alveolar macrophages [30].

Regarding the age effect on seasonal-related CT value, the seasonal differences we observed are consistent with literature: environmental factors interact with host-related factors to shape infection dynamics. On one hand, increased age increases susceptibility to viral infections under any circumstance via impaired particle clearance, cellular senescence, and an increase in permeability of the lungs [16, 37]. On the other hand, cold and rainy seasons introduce reduced social interaction and potential vitamin D deficiency due to decreased sunlight exposure, further compromising immune function [38]. Colder temperatures themselves exert physiological stress, an increase in glucocorticoid and blood glucose [39]. These seasonal factors synergistically interact with age-related vulnerabilities, leading to enhanced viral load observed during cold and rainy seasons.

The opposite, however, was noted inthe seasonal effect on age-related CT values. This observation can be explained by the isolation risks in older adults arising from cognitive decline, limited mobility, and fewer social opportunities [40]. These combined factors likely contribute to a reduction in their overall exposure to potential infectious agents, regardless of the present season. Conversely, children and adults often engage in more frequent social interactions [41] despite a rise in respiratory infections during cold seasons. Consistent with these points, the model’s low explained variance underscores that unmeasured factors likely account for most CT variability, so observed effects should be interpreted as population-level signals rather than individual decision rules.

Overall, there were only minor but steady changes in CT values when we combined sex, age, and season. These minor variations are insufficient to alter care for a single patient and should not be applied as a guide when making decisions. However, even minor changes—particularly seasonal ones—can correspond with variations in the amount of virus in circulation and the number of positive test results observed at the community level. Planning can benefit from that (e.g., staffing, testing capacity, supplies). In line with this notion, our model accounts for only a small portion of the CT ups and downs (R² = 0.019), suggesting that the majority of the variation is likely caused by factors that we were unable to measure such as the number of days following symptoms, the method used to collect the swab, or the specific variant and vaccination status in question. These issues are covered in more detail in the Limitations.

### Limitation

Our study presents several limitations that should be considered when interpreting the findings. Firstly, despite the substantial sample size of 686,739 individuals, it’s crucial to acknowledge that these results were derived solely from data collected at a single center, ALHadi Medical Center, located within the Beirut district. This limited geographic scope may not be fully representative of the entire country, potentially affecting the generalizability of the results to a broader population. Moreover, our study’s timeline is of note, as data collection commenced at the onset of the COVID-19 pandemic in early 2020 and continued through 2023. During this period, PCR assay sensitivity and test kits evolved, which may have introduced heterogeneity in CT values across different phases of the pandemic. This timeframe coincided with a period where a significantly higher number of individuals underwent COVID-19 screening. We did not have variant type or vaccination data, so we couldn’t stratify or adjust for them. Our results reflect the whole study period, and some confounding by waves or vaccine may remain. Furthermore, our findings are based on the categorization of individuals into positive (CT < 35) and negative (CT ≥ 35) groups. It’s important to acknowledge that this CT value cutoff may not be universally applicable and can vary between testing centers or over time, potentially affecting the precision of the results. In addition to the mentioned limitations, it’s crucial to underscore that we did not assess critical factors such as symptoms, comorbidities, chronic medication use, polypharmacy, hospitalizations, ICU admissions, COVID-19-related fatalities, etc. Any mentions of ‘severity’ are contextual background rather than analyses performed in this study. Furthermore, important sampling-related factors such as timing of swab collection in relation to symptom onset, quality of sample collection, and the presence or absence of symptoms at the time of testing were not available in our dataset. These variables are known to influence CT values and may have affected the results. Another limitation is that the season was treated as a binary variable (cold/rainy vs. hot/dry), which may oversimplify the nuanced effects of meteorological conditions. Future studies should integrate continuous day-level data on temperature and humidity from meteorological stations and directly model their association with viral load.

### Recommendations and future studies

To improve representativeness and test reproducibility, multi-center, multi-laboratory studies across diverse Lebanese regions (urban/rural, different socioeconomic strata) are needed. Longitudinal research is essential to track the evolution of COVID-19 testing patterns and their relationship with outcomes over time. Diverse CT value cutoffs should be explored, considering the evolution of testing technologies. Additionally, standardizing CT value reporting across centers and regions is crucial to ensure data consistency.

Future studies should prioritize collecting crucial clinical data (such as symptoms, comorbidities, list of medications, hospitalizations, ICU admissions, or COVID-19-related fatalities) to correlate CT values with symptomatic presentations and the overall disease progression, ultimately providing a more comprehensive picture of the pandemic’s impact on health and healthcare systems. Future studies should also include meteorological station data to obtain continuous variables related to seasonal variation to better capture their influence on viral load dynamics. Furthermore, future studies can investigate the impact of public health interventions on CT values and outcomes, aiding in the evaluation of pandemic control measures.

## Conclusions

It is established based on our study that age, sex, and seasonal fluctuations significantly influence viral load in COVID-19 patients. We found that modest predictors of higher CT values were: younger age, male sex and cold/humid seasons. We also established that in males, CT values were more affected by changes in both age and seasonality, compared to females. However, the maximum impact of sex was seen in older age groups, and was virtually absent in children. Moreover, our study showed that seasonal influences across age and sex suggest that, in hot and dry seasons, the effect of age was lower compared to cold and rainy seasons, maintaining the notion that the magnitude of seasonal influence is highly dependent on age-related factors.

Thus, this research highlights the need to move away from a one-size-fits-all approach to COVID-19 management and embrace personalized strategies that consider sex, age, and seasonal variations. This can ultimately lead to improved patient outcomes, optimized resource utilization, and more effective control of the pandemic.

## Data Availability

The data that support the findings of this study are available from AlHadi Medical Center. Restrictions apply to the availability of these data, which were used under license for this study. Data are available upon request from the corresponding author E.C. with the permission of AlHadi Medical Center.
